# Antimicrobial Evaluation of *Asphodelus microcephalus* Extracts and Fine Powder of Dried Organs Against *Fusarium* and Oomycetes Responsible for Apple and Peach Decline Disease

**DOI:** 10.3390/pathogens14050401

**Published:** 2025-04-22

**Authors:** Sabrine Mannai, Naima Boughalleb-M’Hamdi

**Affiliations:** Department of Biological Sciences and Plant Protection, Higher Institute of Agronomy of Chott Mariem, University of Sousse, L21AGR05, Sousse 4042, Tunisia; sabrinemannai12@gmail.com

**Keywords:** plant extracts, fruit trees seedlings, pathogenic fungi, pseudo-fungi

## Abstract

Seedlings root and collar rot is an important disease that causes a reduction in plant production. The goal of this investigation was to evaluate the in vitro and in vivo efficiency of powdered preparation of dried *Asphodelus microcephalus* of fruits (PPDF), leaves (PPDL), and roots (PPDR) against species of *Fusarium* and Pythiaceaes associated with this disease in Tunisian nurseries. The in vitro tests of methanolic and aqueous extracts of different *Asphodelus* organs showed their efficacy in reducing the pathogen mycelium growth. The in vivo assay of powdered preparation of this dried plant revealed that its effect depends on the pathogens, *Asphodelus* organs, and the period duration between the treatment and inoculation of seedlings. This study showed that this plant has some positive effects, such as disease severity reduction and plant growth stimulation. In fact, for *Fusarium solani*, the powder of different organs of *Asphodelus* was used one week before the plantation, and inoculation significantly improved the peach plant’s height. The treatments eight weeks before the inoculation enhanced the root weight of the plants. Meanwhile, the PPDL and PPDR were used eight weeks before the plantation, and inoculation induced the plant disease index. However, the *A. microcephalus* treatments also have some toxic effects on peach and apple seedlings, such as the improvement of root browning induced by some pathogens.

## 1. Introduction

Apple (*Malus domestica*) and peach (*Prunus persica*) crops occupy an important place throughout the world in terms of total fruit yield in the industry [1]. Several species of oomycetes and fungi like *Pythium ultimum*, *Pythium intermedium*, *Pythium irregulare*, *Ythium heterothallicum*, *Pythium sylvaticum*, and *Pythium vexans* [2,3,4], *Phytopythium mercuriale* [4], *Phytophthora cactorum*, *Phytophthora citrophthora* [4,5], *Cylindrocarpon* spp., *Fusarium oxysporum*, and *Fusarium solani* [6,7,8] revealed to be virulent on apple and peach plants.

Various strategies were employed for the control of fungal infection, ranging from the fumigation with methyl bromide to the management practices. In fact, the fumigation was effective in reducing soilborne inoculum, but, it was removed because of its ozone-depleting effect. However, the management practices have a great impact on quantitative and qualitative soil microbial communities in agricultural ecosystems [9,10].

The protection against plant pathogenic fungi and pseudo-fungi can also be provided by the application of fungicides such as the two fungicides fosetyl-Al and metalaxyl, which have excellent systemic activity against several diseases caused by *Phytophthora* and *Pythium* [2,11]. The effectiveness of chemical protection is not always satisfactory, and its effects on the environment are not negligible. Economic and environmental pressures to reduce reliance on chemical practices have sparked renewed interest in the use of biological methods like bacteria and antagonistic fungi [12], organic soil amendments with, among others, compost [13,14], and plant extracts [15].

Plants can produce compounds called allelochemicals that directly or indirectly affect their biological environment and influence the growth, health, or behavior of other organisms [15,16]. One of the reasons for the interest in allelochemicals is their potential for use in pest control. In fact, several plant compounds like 1,8-dihydroxyanthracene derivatives, flavonoids, phenolic acids, tannins, and triterpenoids possess biological activities such as anti-microbial, anti-fungal and anti-parasitic [17,18,19,20]. Thus, the use of plant allelochemicals in agricultural and horticultural practices could reduce the use of synthetic pesticides, potentially associated with environmental contamination, and contribute to a more sustainable agricultural system. However, there are some unuseful plants, including trees, that are serious pests that can cause damage to most crops. Some of these trees inhibit seed germination and growth of other plants by means of producing toxic allelochemicals [21,22].

In Tunisia, Mannai et al. [15] demonstrated the efficacy of the organic and aqueous extracts and the fine powder of *Raphanus raphanistrum* dried against *Fusarium* and *Pythiaceaes* species associated with apple and peach seedling decline in Tunisian nurseries. This investigation revealed that glucosinolate products and thiocyanate molecules are responsible for antimicrobial activities.

The genus *Asphodelus* is one of the most used medicinal plants. Several studies of the biological activities of different species of *Asphodelus* extracts have shown the antimicrobial activities of this plant [23,24,25].

Immediate studies and research are needed to determine the behavior of biological management measures of significant peach and apple decline in Tunisian nurseries. The present research was conducted in order to study the in vitro and in vivo effectiveness of the extracts and fine powder of dried organs of *Asphodelus microcephalus*, a plant that grows spontaneously in Tunisia, in the management of species of *Fusarium* and Pythiaceae causing apple and peach seedling decline disease.

## 2. Materials and Methods

### 2.1. Pathogens Used

Two isolates of *Fusarium* (*F. oxysporum*, *F. solani*), one isolate of *Pythium* (*P. ultimum*), one isolate of *Phytopythium* (*P. mercuriale*), and one isolate of *Phytophthora* (*P. citrophthora*) were used in this study (Table 1). The tested isolates were obtained from symptomatic peach and apple seedlings grown in Tunisian nurseries. These isolates were earlier proved to be causative agents of the decline of apples and peaches [4,7].

### 2.2. Characteristics of Asphodelus microcephalus

**Figure 1:**
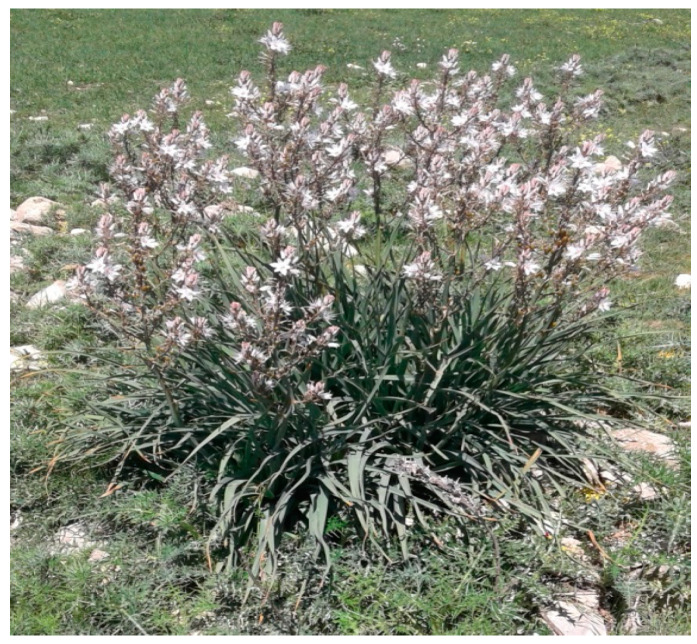
General aspect of *Asphodelus microcephalus*.

The studied plant *Asphodelus microcephalus* (Figure 1) was harvested in April 2016 from the region of Nebeur (Kef).

### 2.3. Preparation of Extracts

The freshly harvested plants were washed and dried in the shade in a dry and ventilated place. Subsequently, the parts used (leaves, immature fruits, and roots were each used alone) were crushed with a fine mesh electric grinder. The powdered preparation of dried *Asphodelus microcephalus* of fruits (PPDF), leaves (PPDL), and roots (PPDR) will be used for biocontrol trials.

#### 2.3.1. Preparation of Aqueous Extracts

To prepare an aqueous extract, a sample of 50 g of powder was placed in a glass jar and macerated in 200 mL of sterile distilled water for 24 h at room temperature. The aqueous phase of the macerate was then filtered through a sterile microfilter (0.22 µm) to avoid contamination.

#### 2.3.2. Preparation of Methanolic Extracts

The partially modified method of Javaid and Bashir [26] was followed to prepare the methanolic extracts. Thus, a quantity of 20 g of powder from each plant organ was packed in a sterile filter paper case and was extracted with methanol 100% (maceration in 500 mL of methanol for one week). The extracts were concentrated in a rotary evaporator (Heidolph Instruments, Schwabach, Germany) at 55 °C. After concentration, these extracts were dried in the open air for some days, then stored at 4 °C, protected from light until use. The methanolic extract yield was calculated according to the following formula:

Y = ((O − E)/O) × 100

with O: the weight of the powdered preparation of dried organ (leaves/roots/fruits) used, E: the weight of the dried obtained methanol extract.

### 2.4. Effect of Aqueous Extracts on Mycelial Growth of Pathogens Associated with Apple and Peach Plants

To study the effect of plant extracts on the mycelial growth of different pathogens, the in vitro poisoned food technique was used according to the methodology of Mazzola et al. [2] with some modifications. This technique consists of adding the extracts to Erlenmeyer flasks containing the PDA medium (45 °C) at different concentrations (5, 10, and 15% *v*/*v*) and then pouring the mixture into Petri dishes. For the control, the same volume of sterile distilled water was used in place of the extract. Then, a disc of the pathogen 0.6 cm in diameter was placed in the center of each dish. Three replicates were performed for each concentration and for each isolate, and the assay was repeated twice. The Petri dishes were incubated at 25 °C for 3 days for *P. ultimum* and *P. mercuriale* and 6 days for *F. oxysporum*, *F. solani*, and *Ph. Citrophthora*. The percentage of growth inhibition (PI) relative to the control was calculated according to the following formula:

PI = ((C − T)/C) × 100

with T: Mean diameter of the colonies in the presence of the plant extract, C: Control colonies Mean diameter.

### 2.5. Effect of Methanolic Extracts on Mycelial Growth of Pathogens Associated with Apple and Peach Plants

The method of Oumzil et al. [27] was followed to study the effect of methanolic extracts on pathogens. This method consists of placing an agar disc 6 mm in diameter, obtained from an approximately seven-day culture of each pathogen, along a diametral axis at a distance of 3 cm from a well (6 mm in diameter) inoculated with 20 μL of the extract to be tested. In untreated control cultures, the methanolic extract was replaced with 20 µL of methanol. Three repetitions were performed for each extract and for each isolate, and the assay was repeated twice. The petri dishes were incubated at 25 °C for 5 days. The percentage inhibition (PI) was calculated according to the following formula:

PI = (1 − T/C) × 100

with T: Average radius of the colonies in the presence of the plant extract, C: Mean radius of control colonies.

### 2.6. Effect of Powdered Preparation of Dried A. microcephalus Organs on Disease Severity

The methodology of Mazzola and Zhao [28] was followed to test the antifungal effect of the three organs of *Asphodelus* plants in vivo. This method consists of treating the substrate (50% sterilized soil, 25% sterilized peat, and 25% sand) with 1% of *A. microcephalus* organs fine powder and incubating it for 24 h in a closed plastic bag. Then, the substrate was divided into two parts. The first part of the soil was put in open containers for a week at room temperature. The second part was moved to open trays at room temperature for 8 weeks. Afterward, four-week-old apple and peach seedlings, ‘MM106 and Garnem’, respectively, were transplanted into the treated substrate. The seedlings were grown in a greenhouse in pots (23 cm diameter × 23 cm deep).

The inoculum for each oomycete isolate was prepared by growing the pathogen in 1000 mL flasks containing 200 g of sand, 20 g of oat, and 30 mL of distilled water, which had been autoclaved twice at 120 °C for 20 min. This mixture was inoculated with 10 agar discs (6 mm diameter) of each oomycete isolate per flask under aseptic conditions. To prepare the *Fusarium* inoculum, bottles containing 200 g of sterile wheat were inoculated with 10 mycelial discs of each *Fusarium* isolate grown on PDA medium for two weeks. Wheat and sand-oat were inoculated with discs of PDA medium, which served as controls. The flasks were shaken to mix the contents and incubated for one week for oomycetes and two weeks for *Fusarium* at 25 °C and shaken every three days to guarantee the colonization. Sand-oat and wheat inoculums were added to the potting mix at a rate of 1% (*v*/*v*), which was then placed in 23 cm diameter plastic pots. The uninoculated control was an uninfested potting mix.

The experiment was carried out according to a completely random arrangement, with three repetitions per treatment. The uprooting took place three months after inoculation. Four parameters were noted before and after the plants’ uprooting (disease index, height, root weight, and root browning index). The disease index of the vegetative part of plants is rated on a 0–5 scale, where 0 = no obvious symptoms; 1 = moderate discoloration of plant leaves (≤25%); 2 = moderate discoloration and falling leaves (26–50%); 3 = moderate discoloration of plant collar, stem and leaves (51–75%); 4 = extensive discoloration of plant collar and stem with falling leaves (>75%); and 5 = dead plant [29]. The root browning index was also rated on a 0–5 scale (0 = no obvious symptoms; 1 = moderate discoloration of root tissue; 2 = moderate discoloration of tissue with some lesion; 3 = extensive discoloration of tissue; 4 = extensive discoloration of tissue with girdling lesions; and 5 = dead plant) [5]. Subsequently, re-isolation from the roots of different plants was carried out using a PDA medium and a PARP-BH medium.

### 2.7. Statistical Analysis

Statistical analyses (ANOVA) were performed for the in vitro and in vivo assays following a completely randomized factorial design. The *A. microcephalus* organ extracts and the pathogen species (methanolic extracts test) or the treatment doses (aqueous extracts test) were the two fixed factors for the in vitro tests. The plant organs used, the period duration between the treatment and inoculation of seedlings and the pathogen species were the three fixed factors for the in vivo tests. Three replicates were used per individual treatment. Data analyses were performed using SPSS Software version 23 (IBM SPSS, Armonk, NY, USA), and mean separations were carried out using the S–N–K test (*p* < 0.05).

## 3. Results

### 3.1. Methanolic Extracts Yield

The results showed a difference in the yields of methanolic extract between the different organs of *Asphodelus*. Indeed, the best yield (37.8%) was obtained for the extract of the leaves, followed by that of the roots (27.35%) and then the fruit extracts (6.33%) (Table 2).

### 3.2. Effect of Asphodelus microcephalus Aqueous Extracts on the Mycelial Growth of Pathogens Associated with Apple and Peach Plants Decline

The analysis of the results showed that the two studied factors (extracts and doses), considered independently or in interaction, had a highly significant effect (*p* ≤ 0.001) on the inhibition percentage of mycelial growth of the pathogens studied (Supplementary Tables).

All extract doses inhibited the mycelial growth of pathogens. In fact, the inhibition percentage of *F. oxysporum* mycelial growth was 100% for all treatments except the extract of the fruits of *A. microcephalus* applied at 5%, where the inhibition was 72.73%. In the case of *F. solani*, the different doses of aqueous extracts from the different organs of *A. microcephalus* completely inhibited mycelial growth (100%). In the case of *Phytophthora citrophthora*, the different doses of the aqueous leaf extract completely inhibited the mycelial growth of the pathogen (100%).

**Figure 2:**
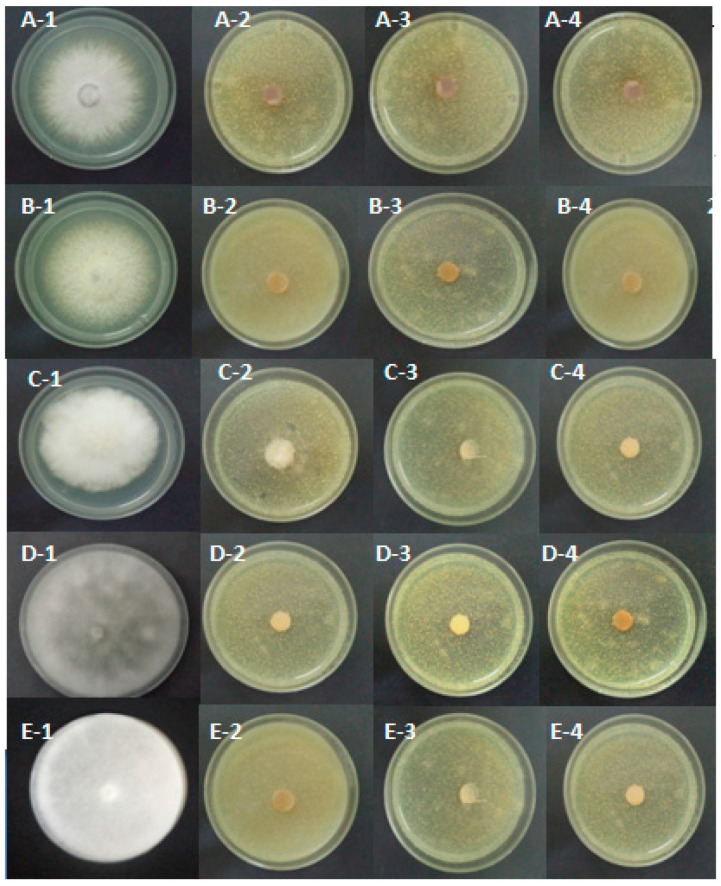
Inhibition of mycelial growth of *Fusarium oxysporum* (**A**), *F. solani* (**B**), *Phytophthora citrophthora* (**C**), *Pythium ultimum* (**D**) and *Phytopythium mercuriale* (**E**) in the presence of 15% of the aqueous extracts of *Asphodelus microcephalus* organs tested, recorded after four days of incubation at 25 °C. 1: control colonies, 2: *Asphodelus* fruits, 3: *Asphodelus* roots, 4: *Asphodelus* leaves.

Extracts from fruits also completely inhibited the mycelial growth of the pathogen at 10 and 15% doses. The extract from the roots was shown to be the least effective against *Phytophthora citrophthora*, with inhibition percentages of 69.19%, 73.51%, and 77.84% at 5%, 10%, and 15% doses, respectively. In the case of *P. ultimum*, the different doses of the aqueous extract of the leaves and the roots of *A. microcephalus* completely inhibited mycelial growth (100%). Whereas, fruit extracts inhibited *P. ultimum* mycelial growth by 100% at 10 and 15% doses. In the case of *P. mercuriale*, the different doses of the aqueous extract of the leaves and roots of *A. microcephalus* completely inhibited the pathogen mycelial growth (100%). However, the fruit extract inhibited *P. mercuriale* mycelial growth by 100% at doses of 10% and 15% (Figure 2 and Table 3).

### 3.3. Effect of Asphodelus microcephalus Methanolic Extracts on the Mycelial Growth of Pathogens Associated with Apple and Peach Plants Decline

**Figure 3:**
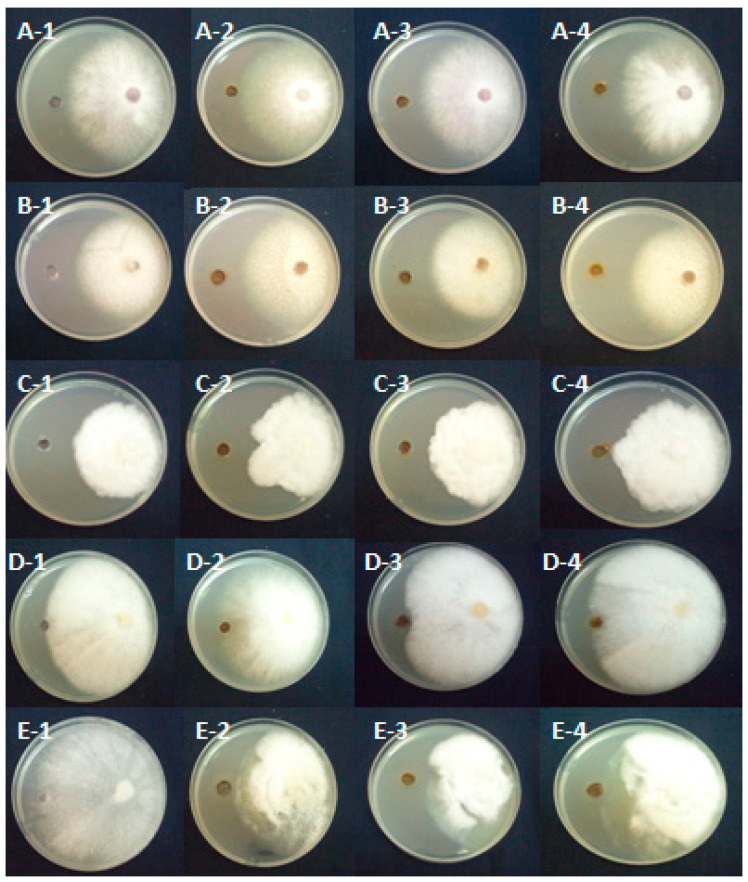
Inhibition of mycelial growth of *Fusarium oxysporum* (**A**), *F. solani* (**B**), *Phytophthora citrophthora* (**C**), *Phytopythium mercuriale* (**D**), and *Pythium ultimum* (**E**) in the presence of methanolic extracts from *Asphodelus microcephalus* parts, recorded after five days of incubation at 25 °C. 1: control colonies, 2: *Asphodelus* leaves, 3: *Asphodelus* fruits, 4: *Asphodelus* roots.

A highly significant interaction was observed between both fixed factors (pathogen isolates and extracts) at *p* ≤ 0.001. In fact, the results of this study showed that the two treatments, PPDL and PPDR, were more effective than PPDF in reducing the mycelial growth of *F. oxysporum* by 3.72% and 3.72%, respectively. The effect of methanolic extracts on the mycelial growth of *P. citrophthora* revealed that the PPDL extract was shown to be more effective than the other extracts. In fact, its inhibition was 26.39%, followed by 19.03% for PPDF extract. The PPDL extract was also the most effective against *Pythium ultimum*, with a mycelial growth inhibition percentage of 23.83%, followed by the PPDR extract (14%). The methanolic extracts of PPDF were more effective against *P. mercuriale* than the other extracts, with an inhibition percentage of 20.22%. However, the three extracts didn’t reduce the mycelial growth of *F. solani* (Figure 3 and Table 4).

### 3.4. Effect of Asphodelus microcephalus In Vivo on Inoculated Peach Seedlings

Variance Analysis of obtained results showed that the plant organs, the period duration between the treatment and inoculation of seedlings, and the pathogen species factors, considered independently or in interaction, had a significant effect (*p* ≤ 0.05) on the disease severity indexes and growth parameters of peach plants.

For *F. solani*, the PPDF, PPDL, and PPDR used before one week significantly improved plant height by 50%, 46.88%, and 21.19%, respectively. The treatment before eight weeks significantly enhanced the root weight of the plants by 135.63%, 264.75%, and 190.80% for PPDF, PPDL, and PPDR, respectively (Table 5).

However, there is a negative (toxic) effect on peach seedlings. In fact, the treatment before 1 week with PPDR improved the root browning index and the disease index of plants inoculated by *F. solani*. The PPDL increased the disease index of seedlings inoculated by *F. solani* (Table 5).

In the case of *F. oxysporum*, the use of PPDL significantly increased the height and the root weight when it has been used before one week by 15.77% and 191.67%, respectively, and decreased the disease index of the plants by 74.91% when this treatment was before eight weeks of inoculation. The PPDF used before one and eight weeks increased the root weight by 230.9% and 203.13%, respectively. Besides, the use of PPDR before one week also improved the root weight by 160.42% (Table 5).

On the other hand, it has a harmful effect on peach seedlings. In fact, the treatment before 1 week with PPDR improved the root browning index of peach seedlings inoculated by *F. oxysporum* (Table 5).

For peach seedlings inoculated by *P. ultimum*, the PPDR, used one week before the inoculation and planting, significantly improved root weight by 92.18% compared to the inoculated control. The use of PPDF before eight weeks boosted this parameter by 107.28% compared to the inoculated control. However, there is a toxic effect on peach seedlings for some treatments. In fact, the treatment before 1 week with PPDR and PPDL before 8 weeks of the inoculation improved the disease index of plants inoculated by *P. ultimum* by 79.64%. The use of PPDF and PPDR before 8 weeks reduced the height of seedlings inoculated by *P. ultimum* (Table 6).

For peach seedlings inoculated by *P. citrophthora*, the PPDF used before one week and eight weeks stimulated the plant’s height significantly by 33.01% and 30.8% and the root weight by 180.31% and 177.23%, respectively, compared to the inoculated control. However, the treatments one week before PPDL and PPDR significantly increased the root weight, respectively, by 205.23% and 118.46%, compared to the inoculated control. For disease index, the treatment before eight weeks of planting and inoculation, with PPDF was more effective than before one week. Results also showed that the treatments before one week with PPDL and PPDR significantly increased the root browning index compared to the inoculated control (Table 6).

### 3.5. Effect of Asphodelus microcephalus In Vivo on Inoculated Apple Seedlings

The results of the effect of powdered preparation of different dried *A. microcephalus* organs on apple seedling decline severity revealed a significant effect of the pathogen species, a highly significant effect of the period duration between the treatment and inoculation of seedlings and the organ used (*p* ≤ 0.001) and significant (*p* ≤ 0.05) interactions (period duration between the treatment and inoculation of seedlings × pathogen species) and (organ used × pathogen species) on the root browning and disease indexes, seedlings height and root weight noted three months after the inoculation. In fact, the PPDF, PPDL, and PPDR used 8 weeks before the apple seedlings inoculation by *P. ultimum* improved the root weight by 97.73, 129.5, and 89.93%, respectively, than the inoculated control. However, the PPDR treatment 1 week and 8 weeks before the inoculation by *P. mercuriale* significantly reduced the apple seedlings’ root browning index by 62.55%. The use of different *Asphodelus* organs one week before the inoculation by *P. ultimum* significantly reduced the seedling’s height. Besides, these treatments reduced the same parameters for the two periods of duration between the treatment and inoculation of seedlings with *P. mercuriale.* The PPDF also reduced the root weight compared to the control inoculated by *P. mercuriale* (Table 7).

## 4. Discussion

The test of aqueous and methanolic extracts from different parts of the *A. microcephalus* plant in vitro showed their effectiveness in inhibiting the mycelial growth of the pathogens used. The three doses (5, 10, and 15%) of the three *A. microcephalus* aqueous extracts were able to inhibit the in vitro mycelia growth of *F. oxysporum*, *F. solani*, *P. ultimum*, *P. citrophthora*, and *P. mercuriale.* These results are supported by the findings of other authors [20], who reported the inhibitory effect of aqueous extract of *Asphodelus tenuifolius* in vitro on two phytopathogenic fungi ‘*Fusarium graminearum* and *F. sporotrichioides*’. The extract strongly inhibited the growth of *F. graminearum* mycelium by 56.89 and 60.34% at 10 and 20% extract concentrations, respectively. Chemical characterization showed the presence of polyphenols in the form of flavonoids and tannins [19].

Recently, Zöngür and Buzpinar [30] tested Seven doses (1, 5, 10, 15, 20, 25, 30 mg/mL) of methanolic *A. aestivus* leaf extract in vitro against *Aspergillus flavus* and *Aspergillus parasiticus* and showed a significant decrease in the mycelia growth diameter. The inhibition rates were recorded between 3.13 and 89.94%. The inhibition effects were 65.96% and 61.22% at 30 mg/mL, against *A. flavus* and *A. parasiticus* respectively.

The effect of *A. microcephalus* in vivo varied depending on the organs used and the period duration between the treatment and inoculation of seedlings and pathogens. The treatment of the substrate with PPDF, PPDL, and PPDR before one and eight weeks had some beneficial effects. In fact, for *F. solani*, the extracts of different organs of *Asphodelus were* used one week before the plantation, and inoculation significantly improved the peach plant height. The use of the three treatments before eight weeks significantly improved the peach root weight. Meanwhile, the PPDL and PPDR used before eight weeks significantly improved the disease index of peach plants. In the case of *F. oxysporum*, the use of PPDL significantly improved the height and the root weight when it was used before one week and the disease index when the treatment was before 8 weeks of inoculation. The PPDF used before one and eight weeks increased the peach root weight. Besides, the use of PPDR before one week also improved the same parameter. For peach seedlings inoculated by *P. ultimum*, the PPDR, used one week before inoculation and planting, significantly improved root weight. The use of PPDF before eight weeks boosted this parameter. For peach seedlings inoculated by *P. citrophthora*, the treatments before one week and before eight weeks with PPDF stimulated the height of the plants and the root weight. The treatments before one week with PPDL and PPDR significantly increased the root weight. The treatment before eight weeks was more effective than before one week, which improved the disease index. The PPDF, PPDL, and PPDR used 8 weeks before the apple seedlings inoculation by *P. ultimum* improved the root weight. However, the treatment by PPDR, 1 week and 8 weeks before the inoculation by *P. mercuriale*, significantly reduced the apple seedlings’ root browning index. Previous studies have shown that 1,8-dihydroxyanthracene derivatives, flavonoids, phenolic acids, and triterpenoids were the main classes of compounds identified in the roots, leaves, and fruits of *Asphodelus* species, which correlate with their biological activities such as anti-microbial, anti-fungal and anti-parasitic [18]. The root tuber part mainly contained anthraquinone derivatives, triterpenoids, and naphthalene derivatives, while the aerial parts mainly exhibited the presence of flavonoids, phenolic acids, and some anthraquinones. Considering previous phytochemical studies, 1,8-dihydroxyanthracene derivatives are the most frequently reported anthraquinones from extracts of *A. aestivus*, *A. luteus*, and *A. microcarpus* that may be responsible for the reported antimicrobial activities [17,18]. This may explain the effectiveness of the root part of *Asphodelus* tested in our study.

However, the results also showed that some treatments had a detrimental effect on apple and peach seedlings. In fact, the treatment before 1 week with PPDR improved the root browning index of peach seedlings inoculated by *F. oxysporum*, *F. solani*, and *P. citrophthora* and the disease index of plants inoculated by *F. solani* and *P. ultimum*. The PPDL stimulated the root browning induced by *P. citrophthora* and the disease index of seedlings inoculated by *F. solani* and *P. ultimum*. The application of PPDL before 8 weeks of inoculation by *P. ultimum* improved the disease index. The use of PPDF and PPDR before 8 weeks reduced the height of seedlings inoculated by *P. ultimum*. These results are supported by previous findings, proving that several allelochemicals impose potential phytotoxicity in other plants, affecting growth, survival, and reproduction [31,32,33]. In addition, previous studies showed that allelochemicals produced by plants interfered with the surrounding environment of other plants, which in turn resulted in alteration in soil biotic factors like soil microbial activity or abiotic factors like soil acidity and fertility, therefore playing a significant role in agroecosystems [34,35].

These results are in agreement with a previous study designed to evaluate the allelopathic effect of the extracts from the roots, stems, fruits, and soil beneath *A. tenuifolius* plants on the seed germination and seedling growth of chickpea showing that the soil beneath the *A. tenuifolius* plants significantly reduced the emergence, root length, shoot length, shoot dry weight, and seedling dry weight but increased the root dry weight of the chickpea seedlings, which suggested that *A. tenuifolius* releases phytotoxic compound (s) [21]. Different concentrations of *A. tenuifolius* shoot extract and seed extract both significantly inhibited the germination and growth behavior of Maize and Sorghum seedlings [36]. Several studies indicated that different parts of the same allelopathic plants might possess different phytotoxic effects on the growth and development of recipient plants [37,38]. Besides, previous studies documented that some allelochemicals enhanced protein and chlorophyll degradation in recipient plants. Moreover, some phenolic acids were reported to inhibit the incorporation of certain amino acids into proteins and thus reduce the rate of protein synthesis [39,40,41,42].

## 5. Conclusions

In the presented study, the results demonstrate the antimicrobial potential of the PPDF, PPDL, and PPDR aqueous and methanolic extracts of *A. microcephalus* against apple and peach decline pathogens in vitro. The in vivo tests of PPDF, PPDL, and PPDR show that their effects depend on the pathogen, the host plant, the organ used, and the period duration between the treatment and inoculation. Some treatments reduced the disease and root browning indexes induced by some pathogens and/or improved the seedlings’ growth (height and root weight). However, it has been concluded that the selected plant for these experiments may also affect the growth behavior in inhibitory ways and the disease severity in stimulatory ways. So, it will be important to study the chemical composition of this plant to determine its beneficial and toxic allelochemicals.

## Figures and Tables

**Table 1:** Pathogen isolates used in this investigation.

Species	Isolates	Origins	Sampling Years	GenBank Accession Number
*F. oxysporum*	Fo22	peach	2013	MF993097
*F. solani*	F48	peach	2012	MF993094
*Pythium ultimum*	P42	peach	2013	MF993110
	Po2	apple	2012	MH260594
*Phytopythium mercuriale*	Po26	apple	2013	MF993112
*Phytophthora citrophthora*	P39	peach	2013	ND

ND: Not determined.

**Table 2:** Methanolic extract yield of *Asphodelus microcephalus* organs studied.

Organs	Methanolic Extract Yield (%)
Fruits	6.33
Leaves	37.80
Roots	27.35

**Table 3:** Percentage inhibition of the mycelial growth of *Fusarium oxysporum*, *F. solani*, *Phytophthora citrophthora*, *Pythium ultimum*, and *Phytopythium mercuriale* in the presence of different doses of aqueous extracts from the plants tested.

Pathogens	Doses (%)	Fruits	Leaves	Roots	*p*-Value ***
*Fusarium solani*	5	100.00 ± 0.00 ^aA^*	100.00 ± 0.00 ^aA^	100.00 ± 0.00 ^aA^	
	10	100.00 ± 0.00 ^a^**^A^	100.00 ± 0.00 ^aA^	100.00 ± 0.00 ^aA^	
	15	100.00 ± 0.00 ^aA^	100.00 ± 0.00 ^aA^	100.00 ± 0.00 ^aA^	
*p*-value					
*Fusarium oxysporum*	5	72.73 ± 0.06 ^aA^	100.00 ± 0.00 ^aB^	100.00 ± 0.00 ^aB^	*p* ≤ 0.05
	10	100.00 ± 0.00 ^bA^	100.00 ± 0.00 ^aA^	100.00 ± 0.00 ^aA^	
	15	100.00 ± 0.00 ^bA^	100.00 ± 0.00 ^aA^	100.00 ± 0.00 ^aA^	
*p*-value		*p* ≤ 0.05			
*Phytophthora citrophthora*	5	51.35 ± 2.79 ^aA^	100.00 ± 0.00 ^aB^	69.19 ± 2.07 ^aA^	*p* ≤ 0.05
	10	100.00 ± 0.00 ^bB^	100.00 ± 0.00 ^aB^	73.51 ± 1.08 ^bA^	*p* ≤ 0.05
	15	100.00 ± 0.00 ^bB^	100.00 ± 0.00 ^aB^	77.84 ± 2.07 ^cA^	*p* ≤ 0.05
*p*-value		*p* ≤ 0.05			
*Pythium ultimum*	5	75.00 ± 1.57 ^aA^	100.00 ± 0.00 ^aB^	100.00 ± 0.00 ^aB^	*p* ≤ 0.05
	10	100.00 ± 0.00 ^bA^	100.00 ± 0.00 ^aA^	100.00 ± 0.00 ^aA^	
	15	100.00 ± 0.00 ^bA^	100.00 ± 0.00 ^aA^	100.00 ± 0.00 ^aA^	
*p*-value		*p* ≤ 0.05			
*Phytopythium mercuriale*	5	80.19 ± 1.09 ^aA^	100.00 ± 0.00 ^aB^	100.00 ± 0.00 ^aB^	*p* ≤ 0.05
	10	100.00 ± 0.00 ^bA^	100.00 ± 0.00 ^aA^	100.00 ± 0.00 ^aA^	
	15	100.00 ± 0.00 ^bA^	100.00 ± 0.00 ^aA^	100.00 ± 0.00 ^aA^	
*p*-value		*p* ≤ 0.05			

(*) Means ± standard error in a row followed by the same capital letter (A, B) are not significantly different according to the SNK test at *p* ≤ 0.05. (**) For each pathogen, means ± standard error in the column followed by the same lowercase letter (a, b, c) are not significantly different according to the SNK test at *p* ≤ 0.05. (***) Probabilities associated with individual F tests. *p*-values listed in the last column refer to all extracts for the dose in that row. *p*-values listed in the last row for each pathogen refer to all three doses for the extract in that column.

**Table 4:** Percentage inhibition of the mycelial growth of *Fusarium oxysporum*, *F. solani*, *Phytophthora citrophthora*, *Pythium ultimum*, and *Phytopythium mercuriale* in the presence of methanolic extracts from *Asphodelus microcephalus* recorded after five days of incubation at 25 °C.

	*Fusarium oxysporum*	*F. solani*	*Phytophthora citrophthora*	*Pythium ultimum*	*Phytopythium mercuriale*	*p*-Value ***
PPDF	0.00 ± 0.00 ^A^*^a^**	0.00 ± 0.00 ^Aa^	19.03 ± 7.94 ^Bb^	9.09 ± 2.46 ^Aa^	20.22 ± 5.10 ^Bb^	*p* ≤ 0.001
PPDL	3.72 ± 1.57 ^Bb^	0.00 ± 0.00 ^Aa^	26.39 ± 1.82 ^Db^	23.83 ± 2.46 ^Db^	10.75 ± 2.03 ^Ca^	*p* ≤ 0.001
PPDR	3.72 ± 1.57 ^Ab^	0.00 ± 0.00 ^Aa^	5.36 ± 5.84 ^Aa^	14.00 ± 10.42 ^Ba^	4.67 ± 2.03 ^Aa^	*p* ≤ 0.05
*p*-value ***	*p* ≤ 0.05	*p* ≥ 0.05	*p* ≤ 0.05	*p* ≤ 0.05	*p* ≤ 0.05	

(*) Means ± standard error in a row followed by the same capital letter are not significantly different according to the SNK test at *p* ≤ 0.05. (**) Means ± standard error in the column followed by the same lowercase letter are not significantly different according to the SNK test at *p* ≤ 0.05. (***) Probabilities related to individual F tests. All five pathogens for the extract in that row are referenced by the *p*-value in the final column. The *p*-value indicated in the final row pertains to every extract for the pathogen in that particular column. PPDF: a powdered preparation of dried fruits; PPDR: a powdered preparation of dried roots; PPDL: a powdered preparation of dried leaves.

**Table 5:** Effect of *Asphodelus microcephalus* fruit, leaf, and root fine powders on the severity of *Fusarium oxysporum* and *F. solani* on peach seedlings ‘Garnem’ and the growth parameters recorded three months after the inoculation.

Pathogens	Parameters	Treatment	Fruits	Leaves	Roots	*p*-Value ***
*F. oxysporum*	Root Browning Index	1W	2.00 ± 0.00 ^a^*^A^**	1.67 ± 0.58 ^aA^	2.67 ± 0.58 ^bA^	*p* ≥ 0.05
		8W	1.67 ± 0.58 ^aA^	1.67 ± 0.58 ^aA^	2.00 ± 0.00 ^abA^	*p* ≥ 0.05
		NIC	2.00 ± 0.00 ^a^	2.00 ± 0.00 ^a^	2.00 ± 0.00 ^a^	nd
		IC	1.33 ± 0.43 ^a^	1.33 ± 0.43 ^a^	1.33 ± 0.43 ^a^	nd
	*p*-value		*p* ≥ 0.05	*p* ≥ 0.05	*p* ≤ 0.05	
	Disease Index	1W	1.67 ± 0.58 ^aA^	1.33 ± 0.58 ^abA^	2.33 ± 0.58 ^aA^	*p* ≥ 0.05
		8W	2.33 ± 0.58 ^aB^	0.67 ± 0.58 ^bA^	3.33 ± 0.58 ^aB^	*p* ≤ 0.05
		NIC	2.33 ± 0.58 ^a^	2.33 ± 0.58 ^a^	2.33 ± 0.58 ^a^	nd
		IC	2.67 ± 0.83 ^a^	2.67 ± 0.83 ^a^	2.67 ± 0.83 ^a^	nd
	*p*-value		*p* ≥ 0.05	*p* ≤ 0.05	*p* ≥ 0.05	
	Height (cm)	1W	65.00 ± 2.00 ^aA^	68.50 ± 8.41 ^bA^	66.50 ± 0.87 ^aA^	*p* ≥ 0.05
		8W	52.83 ± 2.25 ^aB^	39.33 ± 2.52 ^aA^	50.00 ± 3.97 ^aB^	*p* ≤ 0.05
		NIC	62.83 ± 6.79 ^a^	62.83 ± 6.79 ^ab^	62.83 ± 6.79 ^a^	nd
		IC	59.17 ± 13.83 ^a^	59.17 ± 13.83 ^ab^	59.17 ± 13.83 ^a^	nd
	*p*-value		*p* ≥ 0.05	*p* ≤ 0.05	*p* ≥ 0.05	
	Root Weight (g)	1W	9.53 ± 1.14 ^bA^	8.74 ± 2.81 ^bA^	7.50 ± 1.38 ^bA^	*p* ≥ 0.05
		8W	8.73 ± 0.91 ^bB^	5.90 ± 0.33 ^abA^	5.04 ± 1.31 ^abA^	*p* ≤ 0.05
		NIC	7.38 ± 2.09 ^b^	7.38 ± 2.09 ^b^	7.38 ± 2.09 ^b^	nd
		IC	2.88 ± 1.52 ^a^	2.88 ± 1.52 ^a^	2.88 ± 1.52 ^a^	nd
	*p*-value		*p* ≤ 0.05	*p* ≤ 0.05	*p* ≤ 0.05	
*F. solani*	Root browning	1W	2.00 ± 0.00 ^aA^	2.67 ± 0.58 ^bAB^	3.33 ± 0.58 ^bB^	*p* ≥ 0.05
		8W	1.33 ± 0.58 ^aA^	1.67 ± 0.58 ^abA^	1.33 ± 0.58 ^aA^	*p* ≥ 0.05
		NIC	1.33 ± 0.58 ^a^	1.33 ± 0.58 ^a^	1.33 ± 0.58 ^a^	nd
		IC	2.00 ± 0.58 ^a^	2.00 ± 0.58 ^ab^	2.00 ± 0.00 ^a^	nd
	*p*-value		*p* ≥ 0.05	*p* ≤ 0.05	*p* ≤ 0.05	
	Disease Index	1W	3.00 ± 1.00 ^aA^	3.67 ± 0.58 ^cA^	3.67 ± 0.58 ^cA^	*p* ≥ 0.05
		8W	1.33 ± 0.58 ^aA^	0.67 ± 0.58 ^aA^	0.67 ± 0.58 ^aA^	*p* ≥ 0.05
		NIC	1.67 ± 0.58 ^a^	1.67 ± 0.58 ^ab^	1.67 ± 0.58 ^ab^	nd
		IC	2.33 ± 0.58 ^a^	2.33 ± 0.58 ^b^	2.33 ± 0.58 ^b^	nd
	*p*-value		*p* ≥ 0.05	*p* ≤ 0.05	*p* ≤ 0.05	
	Height (cm)	1W	72.00 ± 3.50 ^cB^	70.50 ± 3.00 ^bB^	58.17 ± 0.76 ^cA^	*p* ≤ 0.001
		8W	45.83 ± 0.29 ^aC^	41.50 ± 2.29 ^aB^	36.33 ± 2.75 ^aA^	*p* ≤ 0.05
		NIC	62.83 ± 6.79 ^b^	62.83 ± 6.79 ^b^	62.83 ± 6.79 ^c^	nd
		IC	48.00 ± 4.58 ^a^	48.00 ± 4.58 ^a^	48.00 ± 4.58 ^b^	nd
	*p*-value		*p* ≤ 0.05	*p* ≤ 0.05	*p* ≤ 0.05	
	Root Weight (g)	1W	5.21 ± 1.66 ^abA^	4.58 ± 0.58 ^aA^	4.10 ± 0.86 ^aA^	*p* ≥ 0.05
		8W	6.15 ± 0.20 ^bA^	9.52 ± 0.93 ^bB^	7.59 ± 0.91 ^bA^	*p* ≤ 0.05
		NIC	7.38 ± 2.09 ^b^	7.38 ± 2.09 ^b^	7.38 ± 2.09 ^b^	nd
		IC	2.61 ± 0.78 ^a^	2.61 ± 0.78 ^a^	2.61 ± 0.78 ^a^	nd
	*p*-value		*p* ≤ 0.05	*p* ≤ 0.05	*p* ≤ 0.05	

(*) Means ± standard error in the column for each parameter (root browning index, disease index, height, and root weight) followed by the same lowercase letter are not significantly different according to the SNK test at *p* ≤ 0.05. (**) Means ± standard error in a row followed by the same capital letter are not significantly different according to the SNK test at *p* ≤ 0.05. (***) Probabilities associated with individual F tests. (nd) not determined. *p*-values listed in the last column refer to all three organ treatments for the period of duration between the treatment and inoculation of seedlings in that row. *p*-values listed in the last row for each parameter refer to all treatments (1W, 8W, NIC, and IC) for the pathogen in that column. (NIC) uninoculated control; IC: inoculated control; 1W: substrate treatment one week before planting and inoculation; 8W: substrate treatment eight weeks before planting and inoculation.

**Table 6:** Effect of *Asphodelus microcephalus* fine powder on the severity of *P. ultimum* and *Phytophthora citrophthora* on peach seedlings ’Garnem’ and the growth parameters recorded three months after the inoculation.

Pathogens	Parameters	Treatment	Fruits	Leaves	Roots	*p*-Value ***
*P. ultimum*	Root Browning Index	1W	2.33 ± 0.58 ^a^*^A^**	2.00 ± 0.00 ^a^	2.33 ± 0.58 ^a^	*p* ≥ 0.05
		8W	1.67 ± 0.58 ^a^	2.67 ± 0.58 ^a^	2.33 ± 0.58 ^a^	*p* ≥ 0.05
		NIC	2.00 ± 0.00 ^a^	2.00 ± 0.00 ^a^	2.00 ± 0.00 ^a^	nd
		IC	2.00 ± 0.00 ^a^	2.00 ± 0.00 ^a^	2.00 ± 0.00 ^a^	nd
	*p*-value		*p* ≥ 0.05	*p* ≥ 0.05	*P* ≥ 0.05	
	Disease Index	1W	3.00 ± 1.00 ^a^	2.33 ± 0.58 ^ab^	3.00 ± 0.00 ^b^	*p* ≥ 0.05
		8W	1.33 ± 0.58 ^aA^	3.00 ± 0.00 ^bC^	2.00 ± 0.00 ^aB^	*p* ≤ 0.05
		NIC	2.33 ± 0.58 ^a^	2.33 ± 0.58 ^ab^	2.33 ± 0.58 ^ab^	nd
		IC	1.67 ± 0.58 ^a^	1.67 ± 0.83 ^a^	1.67 ± 0.83 ^a^	nd
	*p*-value		*p* ≥ 0.05	*p* ≤ 0.05	*p* ≤ 0.05	
	Height (cm)	1W	68.17 ± 3.25 ^b^	48.00 ± 17.68 ^a^	60.33 ± 2.02 ^b^	*p* ≥ 0.05
		8W	37.38 ± 7.29 ^a^	48.50 ± 3.77 ^a^	37.67 ± 4.75 ^a^	*p* ≥ 0.05
		NIC	62.83 ± 6.79 ^b^	62.83 ± 6.79 ^a^	62.83 ± 6.79 ^b^	nd
		IC	61.17 ± 4.75 ^b^	61.17 ± 4.75 ^a^	61.17 ± 4.75 ^b^	nd
	*p-value*		*p* ≤ 0.001	*p* ≥ 0.05	*p* ≤ 0.001	
	Root Weight (g)	1W	4.17 ± 0.04 ^aA^	3.79 ± 2.00 ^aA^	7.13 ± 0.60 ^bB^	*p* ≤ 0.05
		8W	7.69 ± 0.77 ^bB^	4.27 ± 0.53 ^aA^	3.28 ± 0.44 ^aA^	*p* ≤ 0.001
		NIC	7.38 ± 2.09 ^b^	7.38 ± 2.09 ^a^	7.38 ± 2.09 ^b^	nd
		IC	3.71 ± 0.26 ^a^	3.71 ± 0.26 ^a^	3.71 ± 0.26 ^a^	nd
	*p*-value		*p* ≤ 0.05	*p*≥ 0.05	*p* ≤ 0.05	
*Ph. citrophthora*	Root Browning Index	1W	3.00 ± 1.00 ^aA^	3.33 ± 0.58 ^bA^	4.00 ± 0.00 ^bA^	*p* ≥ 0.05
		8W	1.33 ± 0.58 ^aA^	1.67 ± 0.58 ^aA^	2.33 ± 0.58 ^aA^	*p* ≥ 0.05
		NIC	2.00 ± 0.00 ^a^	2.00 ± 0.00 ^a^	2.00 ± 0.00 ^a^	nd
		IC	2.33 ± 0.58 ^a^	2.33 ± 0.58 ^a^	2.33 ± 0.58 ^a^	nd
	*p*-value		*p* ≥ 0.05	*p* ≤ 0.05	*p* ≤ 0.05	
	Disease Index	1W	2.33 ± 0.58 ^bA^	2.00 ± 0.00 ^aA^	2.67 ± 0.58 ^aA^	*p* ≥ 0.05
		8W	0.67 ± 0.58 ^aA^	1.33 ± 0.58 ^aA^	1.67 ± 0.58 ^aA^	*p* ≥ 0.05
		NIC	2.33 ± 0.58 ^b^	2.33 ± 0.58 ^a^	2.33 ± 0.58 ^a^	nd
		IC	2.33 ± 0.58 ^b^	2.33 ± 0.58 ^a^	2.33 ± 0.58 ^a^	nd
	*p*-value		*p* ≤ 0.05	*p* ≥ 0.05	*p* ≥ 0.05	
	High (cm)	1W	69.83 ± 6.01 ^bA^	58.83 ± 2.25 ^bA^	62.17 ± 19.04 ^bA^	*p* ≥ 0.05
		8W	68.67 ± 4.25 ^bB^	29.67 ± 2.75 ^aA^	27.67 ± 1.61 ^aA^	*p* ≤ 0.001
		NIC	62.83 ± 6.79 ^a^	62.83 ± 6.79 ^b^	62.83 ± 6.79 ^b^	nd
		IC	52.50 ± 7.26 ^a^	52.50 ± 7.26 ^b^	52.50 ± 7.26 ^b^	nd
	*p*-value		*p* ≤ 0.05	*p* ≤ 0.05	*p* ≤ 0.05	
	Root Weight (g)	1W	9.11 ± 3.16 ^bA^	9.92 ± 1.23 ^cA^	7.10 ± 1.08 ^bA^	*p* ≥ 0.05
		8W	9.01 ± 0.97 ^bC^	4.47 ± 0.51 ^aB^	1.94 ± 0.38 ^aA^	*p* ≤ 0.001
		NIC	7.38 ± 2.09 ^b^	7.38 ± 2.09 ^b^	7.38 ± 2.09 ^b^	nd
		IC	3.25 ± 0.36 ^a^	3.25 ± 0.36 ^a^	3.25 ± 0.36 ^a^	nd
	*p*-value		*p* ≤ 0.05	*p* ≤ 0.05	*p* ≤ 0.05	

(*) The SNK test at *p* ≤ 0.05 indicates that there is no significant difference between the means ± standard error in the column for each parameter followed by the same lowercase letter. (**) The SNK test indicates that there is no significant difference between means ± standard error that are followed by the same capital letter at *p* ≤ 0.05. (***) Probabilities related to specific F tests. (nd) not determined. All treatments in that row are referenced by the *p*-value in the final column. For each pathogen, the period of duration between the treatment and inoculation in that column is included in the *p*-value for each parameter, which is shown in the last row. (NIC) uninoculated control; IC: inoculated control; 1W: substrate treatment one week before planting and inoculation; 8W: substrate treatment eight weeks before planting and inoculation.

**Table 7:** Effect of *Asphodelus microcephalus* extracts on the severity of *P. ultimum* and *Phytopythium mercuriale* on apple seedlings ‘MM106’ and the growth parameters recorded three months after the inoculation.

Pathogens	Parameters	Treatment	Fruits	Leaves	Roots	*p*-Value ***
*Pythium ultimum*	Root Browning Index	1W	2.00 ± 0.00 ^ab^*^A^**	2.00 ± 0.00 ^abA^	2.33 ± 0.58 ^abA^	*p* ≥ 0.05
		8W	2.00 ± 0.00 ^abA^	1.67 ± 0.58 ^abA^	2.00 ± 0.00 ^abA^	*p* ≥ 0.05
		NIC	1.33 ± 0.58 ^a^	1.33 ± 0.58 ^a^	1.33 ± 0.58 ^a^	nd
		IC	2.67 ± 0.58 ^b^	2.67 ± 0.58 ^b^	2.67 ± 0.58 ^b^	nd
	*p*-value		*p* ≤ 0.05	*p* ≤ 0.05	*p* ≤ 0.05	
	Disease Index	1W	2.33 ± 0.58 ^aA^	2.33 ± 0.58 ^aA^	1.67 ± 0.58 ^aA^	*p* ≥ 0.05
		8W	2.00 ± 0.00 ^aA^	1.67 ± 0.58 ^aA^	1.33 ± 0.58 ^aA^	*p* ≥ 0.05
		NIC	1.33 ± 0.58 ^a^	1.33 ± 0.58 ^a^	1.33 ± 0.58 ^a^	nd
		IC	1.67 ± 0.58 ^a^	1.67 ± 0.58 ^a^	1.67 ± 0.58 ^a^	nd
	*p*-value		*p* ≥ 0.05	*p* ≥ 0.05	*p* ≥ 0.05	
	Height (cm)	1W	30.00 ± 0.50 ^aA^	32.83 ± 3.01 ^aA^	33.90 ± 7.35 ^aA^	*p* ≥ 0.05
		8W	52.00 ± 2.00 ^bA^	53.50 ± 8.00 ^bA^	61.00 ± 8.00 ^bA^	*p* ≥ 0.05
		NIC	90.33 ± 11.02 ^c^	90.33 ± 11.02 ^c^	90.33 ± 11.02 ^c^	nd
		IC	62.00 ± 6.00 ^b^	62.00 ± 6.00 ^b^	62.00 ± 6.00 ^b^	nd
	*p*-value		*p* ≤ 0.05	*p* ≤ 0.05	*p* ≤ 0.05	
	Root Weight (g)	1W	3.74 ± 2.44 ^aA^	5.48 ± 1.18 ^aA^	5.81 ± 2.15 ^aA^	*p* ≥ 0.05
		8W	13.94 ± 3.30 ^bA^	16.18 ± 6.20 ^bA^	13.39 ± 4.67 ^bA^	*p* ≥ 0.05
		NIC	11.81 ± 1.32 ^b^	11.81 ± 1.32 ^ab^	11.81 ± 1.32 ^ab^	nd
		IC	7.05 ± 0.06 ^a^	7.05 ± 0.06 ^a^	7.05 ± 0.06 ^a^	nd
	*p*-value		*p* ≤ 0.05	*p* ≤ 0.05	*p* ≤ 0.05	
*Phytopythium mercuriale*	Root Browning Index	1W	2.33 ± 0.58 ^aB^	2.33 ± 0.58 ^aB^	1.00 ± 0.00 ^aA^	*p* ≤ 0.05
		8W	1.67 ± 0.58 ^aA^	1.33 ± 0.58 ^aA^	1.00 ± 0.00 ^aA^	*p* ≥ 0.05
		NIC	1.33 ± 0.58 ^a^	1.33 ± 0.58 ^a^	1.33 ± 0.58 ^a^	nd
		IC	2.67 ± 0.58 ^a^	2.67 ± 0.58 ^a^	2.67 ± 0.58 ^b^	nd
	*p*-value		*p* ≥ 0.05	*p* ≥ 0.05	*p* ≤ 0.05	
	Disease Index	1W	2.33 ± 0.58 ^aA^	2.00 ± 0.00 ^aA^	1.33 ± 0.58 ^aA^	*p* ≥ 0.05
		8W	1.67 ± 0.58 ^aA^	1.33 ± 0.58 ^aA^	1.33 ± 0.58 ^aA^	*p* ≥ 0.05
		NIC	1.33 ± 0.58 ^a^	1.33 ± 0.58 ^a^	1.33 ± 0.58 ^a^	nd
		IC	1.33 ± 0.58 ^a^	1.33 ± 0.58 ^a^	1.33 ± 0.58 ^a^	nd
	*p*-value		*p* ≥ 0.05	*p* ≥ 0.05	*p* ≥ 0.05	
	Height (cm)	1W	38.83 ± 5.13 ^aAB^	44.50 ± 5.27 ^aB^	32.67 ± 1.76 ^aA^	*p* ≤ 0.05
		8W	42.33 ± 8.02 ^aA^	58.33 ± 0.58 ^bA^	59.00 ± 12.00 ^bA^	*p* ≥ 0.05
		NIC	90.33 ± 11.02 ^b^	90.33 ± 11.02 ^c^	90.33 ± 11.02 ^c^	nd
		IC	80.67 ± 2.08 ^b^	80.67 ± 2.08 ^c^	80.67 ± 2.08 ^c^	nd
	*p*-value		*p* ≤ 0.05	*p* ≤ 0.05	*p* ≤ 0.05	
	Root Weight (g)	1W	5.22 ± 0.99 ^aA^	10.04 ± 1.36 ^aB^	8.54 ± 1.89 ^aB^	*p* ≤ 0.05
		8W	6.86 ± 2.71 ^aA^	13.71 ± 3.10 ^aA^	10.97 ± 3.34 ^aA^	*p* ≥ 0.05
		NIC	11.81 ± 1.32 ^b^	11.81 ± 1.32 ^a^	11.81 ± 1.32 ^a^	nd
		IC	11.64 ± 0.45 ^b^	11.64 ± 0.45 ^a^	11.64 ± 0.45 ^a^	nd
	*p*-value		*p* ≤ 0.05	*p* ≥ 0.05	*p* ≥ 0.05	

(*) The SNK test at *p*≤ 0.05 indicates that there is no significant difference between the means ± standard error in the column for each parameter followed by the same lowercase letter. (**) The SNK test indicates that there is no significant difference between means ± standard error that are followed by the same capital letter at *p* ≤ 0.05. (***) Probabilities related to specific F tests. (nd) not determined. *p*-values listed in the last column refer to all three organ treatments for the treatment in the period duration between the treatment and inoculation of seedlings that row. *p*-values listed in the last row for each parameter refer to all treatments for the pathogen in that column. (NIC) uninoculated control; IC: inoculated control; 1W: substrate treatment one week before planting and inoculation; 8W: substrate treatment eight weeks before planting and inoculation.

## Data Availability

The original contributions presented in this study are included in the article. Further inquiries can be directed to the corresponding author.

## Supplementary Materials

The following supporting information can be downloaded at: https://www.mdpi.com/article/10.3390/pathogens14050401/s1, Supplementary Tables: Two and three factors ANOVA tables.
