# Supplementary material for: Antimicrobial Evaluation of Asphodelus microcephalus Extracts and Fine Powder of Dried Organs Against Fusarium and Oomycetes Responsible for Apple and Peach Decline Disease
Source: Pathogens. 2025 Apr 22;14(5):401. doi: 10.3390/pathogens14050401 (PMC12113936; doi:10.3390/pathogens14050401)
Supplement: Supplementary file 1 [file pathogens-14-00401-s001.zip › pathogens-3468632-supplementary.pdf]

1. **Two factors ANOVA tables (The univarate GLM was applied)**

1.1.F. oxysporum

Tests of Between-Subjects Effects

Dependent Variable: Inhibition

Source	Type III Sum of Squares	df	Mean Square	F	Sig.
Corrected Model	2644,628[a]	8	330,579	2187,000	,000
Intercept	338512,397	1	338512,397	2239488,000	,000
Organ	661,157	2	330,579	2187,000	,000
Dose	661,157	2	330,579	2187,000	,000
Organ * Dose	1322,314	4	330,579	2187,000	,000
Error	4,081	27	,151		
Total	341161,106	36			
Corrected Total	2648,709	35			

a. R Squared = ,998 (Adjusted R Squared = ,998)

1.2.P.citrophthora

Tests of Between-Subjects Effects

Dependent Variable: Inhibition

Source	Type III Sum of Squares	df	Mean Square	F	Sig.
Corrected Model	10740,687[a]	8	1342,586	689,250	,000
Intercept	264807,597	1	264807,597	135945,600	,000
Organ	4279,912	2	2139,956	1098,600	,000
Dose	2714,585	2	1357,292	696,800	,000
Organ * Dose	3746,189	4	936,547	480,800	,000
Error	52,593	27	1,948		
Total	275600,877	36			
Corrected Total	10793,280	35			

a. R Squared = ,995 (Adjusted R Squared = ,994)

1.3. P. ultimum

Tests of Between-Subjects Effects

Dependent Variable: Inhibition

Source	Type III Sum of Squares	df	Mean Square	F	Sig.
Corrected Model	2222,222[a]	8	277,778	1014,000	,000
Intercept	340277,778	1	340277,778	1242150,004	,000
Organ	555,556	2	277,778	1014,000	,000
Dose	555,556	2	277,778	1014,000	,000
Organ * Dose	1111,111	4	277,778	1014,000	,000
Error	7,396	27	,274		
Total	342507,396	36			
Corrected Total	2229,619	35			

a. R Squared = ,997 (Adjusted R Squared = ,996)

1.4. *P. mercuriale*

Tests of Between-Subjects Effects

Dependent Variable: Inhibition

Source	Type III Sum of Squares	df	Mean Square	F	Sig.
Corrected Model	1395,514[a]	8	174,439	1323,000	,000
Intercept	344325,383	1	344325,383	2611467,008	,000
Organ	348,879	2	174,439	1323,000	,000
Dose	348,879	2	174,439	1323,000	,000
Organ * Dose	697,757	4	174,439	1323,000	,000
Error	3,560	27	,132		
Total	345724,457	36			
Corrected Total	1399,074	35			

a. R Squared = ,997 (Adjusted R Squared = ,997)

2. Three-factor analysis ((The univarate GLM was applied)

Tests of Between-Subjects Effects

Dependent Variable: Inhibition

Source	Type III Sum of Squares	df	Mean Square	F	Sig.
Corrected Model	21517,539[a]	44	489,035	976,179	,000
Intercept	1643408,666	1	1643408,666	3280461,247	,000
Pathogen	4514,488	4	1128,622	2252,879	,000
Organ	2008,326	2	1004,163	2004,442	,000
Dose	2879,777	2	1439,888	2874,208	,000
Pathogen * Organ	3837,177	8	479,647	957,439	,000
Pathogen * Dose	1400,399	8	175,050	349,423	,000
Organ * Dose	4924,214	4	1231,054	2457,346	,000
Pathogen * Organ * Dose	1953,158	16	122,072	243,673	,000
Error	67,631	135	,501		
Total	1664993,836	180			
Corrected Total	21585,170	179			

a. R Squared = ,997 (Adjusted R Squared = ,996)
